# Comparison of Protective Effects of Polyphenol-Enriched Extracts from Thinned Immature Kiwifruits and Mature Kiwifruits against Alcoholic Liver Disease in Mice

**DOI:** 10.3390/foods13193072

**Published:** 2024-09-26

**Authors:** Wen Deng, Qian-Ni Yang, Ding-Tao Wu, Jie Li, Hong-Yan Liu, Yi-Chen Hu, Liang Zou, Ren-You Gan, Hui-Ling Yan, Jing-Wei Huang

**Affiliations:** 1 Key Laboratory of Coarse Cereal Processing of Ministry of Agriculture and Rural Affairs, School of Food and Biological Engineering, Chengdu University, Chengdu 610106, China; 2 Institute for Advanced Study, Chengdu University, Chengdu 610106, China; 3 Research Center for Plants and Human Health, Institute of Urban Agriculture, Chinese Academy of Agricultural Sciences, National Agricultural Science and Technology Center, Chengdu 610213, China; 4 Department of Food Science and Nutrition, Faculty of Science, The Hong Kong Polytechnic University, Kowloon, Hong Kong, China

**Keywords:** discarded young kiwifruit, phenolic compound, antioxidant effect, alcoholic liver injury, hepatoprotective effects

## Abstract

Alcoholic liver disease (ALD) is regarded as one of the main global health problems. Accumulated evidence indicates that fruit-derived polyphenols can lower the risk of ALD, this attributed to their strong antioxidant capacities. Thinned immature kiwifruits (TIK) are the major agro-byproducts in the production of kiwifruits, which have abundantly valuable polyphenols. However, knowledge about the protective effects of polyphenol-enriched extract from TIK against ALD is still lacking, which ultimately restricts their application as value-added functional products. To promote their potential applications, phenolic compounds from TIK and their corresponding mature fruits were compared, and their protective effects against ALD were studied in the present study. The findings revealed that TIK possessed extremely high levels of total phenolics (116.39 ± 1.51 mg GAE/g DW) and total flavonoids (33.88 ± 0.59 mg RE/g DW), which were about 7.4 times and 4.8 times greater than those of their corresponding mature fruits, respectively. Furthermore, the level of major phenolic components in TIK was measured to be 29,558.19 ± 1170.58 μg/g DW, which was about 5.4 times greater than that of mature fruits. In particular, neochlorogenic acid, epicatechin, procyanidin B1, and procyanidin B2 were found as the predominant polyphenols in TIK. In addition, TIK exerted stronger in vitro antioxidant and anti-inflammatory effects than those of mature fruits, which was probably because of their higher levels of polyphenols. Most importantly, compared with mature fruits, TIK exhibited superior hepatoprotective effects on alcohol-induced liver damage in mice. The administration of polyphenol-enriched extract from TIK (YK) could increase the body weight of mice, reduce the serum levels of ALP, AST, and ALT, lower the levels of hepatic TG and TC, and diminish lipid droplet accumulation and hepatic tissue damage. In addition, the treatment of YK could also significantly restore the levels of antioxidant enzymes (e.g., SOD and CAT) in the liver and lower the levels of hepatic proinflammatory cytokines (e.g., IL-6, IL-1β, and TNF-α), indicating that YK could effectively ameliorate ALD in mice by reducing hepatic oxidative stress and hepatic inflammation. Collectively, our findings can provide sufficient evidence for the development of TIK and their extracts as high value-added functional products for the intervention of ALD.

## 1. Introduction

Alcoholic liver disease (ALD) is induced by long-term and excessive alcohol consumption, which is characterized by a complex spectrum of histological lesions, ranging from steatosis to cirrhosis [1]. It is well established that ALD is associated with oxidative stress and inflammatory reaction [1,2,3]. Oxidative stress is considered one of the most critical factors contributing to ALD, which is triggered by reactive oxygen species (ROS) that are generated during alcohol metabolism [2,4]. ROS can bind to proteins, causing structural and functional alterations and the formation of neoantigens, which can also directly damage DNA or induce lipid peroxidation [4]. Therefore, a reduction in oxidative stress can effectively regulate the development of ALD. In addition, a number of experimental studies have revealed that chronic alcohol consumption can improve the levels of gut microbial-derived lipopolysaccharides (LPS) [5,6]. Gut microbial-derived LPS, as a critical mediator of inflammation in ALD, can activate the TLR 4-NF-κB signal pathway to release proinflammatory cytokines and mediators, ultimately causing liver inflammation [3]. Therefore, the intervention of hepatic inflammation can also positively regulate ALD development. In fact, a large number of studies have demonstrated that phenolic compounds derived from various fruits and vegetables exhibit potential protective effects on alcohol-induced liver damage in mice [4,7]. This is because natural polyphenols from fruits and vegetables are regarded as strong antioxidants, which can alleviate oxidative damage, inflammation, and fat accumulation by chronic alcohol consumption [4,7]. Therefore, the dietary consumption of polyphenol-enriched fruits or vegetables is preferred for intervening in ALD.

Kiwifruit belongs to the genus *Actinidia* in the family Actinidiaceae, which is extremely popular worldwide owing to its delicious taste and outstanding health-promoting effects [8]. Kiwifruit possesses rich phenolic compounds, e.g., phenolic acids, flavanols, flavonols, and anthocyanins, thereby exhibiting strong antioxidant, anti-inflammatory, anti-diabetic, and anti-tumor properties [8]. Generally, to obtain kiwifruits with superior quality and high yield, about 30% of immature kiwifruits are thinned and abandoned in orchards, with these considered the main agro-byproducts in the production of kiwifruits [9]. In fact, compared with mature kiwifruits, these thinned immature kiwifruits (TIK) possess much higher contents of polyphenols, exhibiting superior antioxidant and anti-inflammatory effects [10,11]. These results suggest that TIK may exert potential benefits for the management of ALD owing to their strong antioxidant capacities. Nevertheless, the knowledge about the protective effects of polyphenol-enriched extracts from TIK against ALD in mice is unclear.

Hence, to improve the development and application of TIK as value-added functional products or functional ingredients, phenolic compounds from TIK and their corresponding mature kiwifruits were investigated and compared, and their potential protective effects against the Lieber–DeCarli ethanol liquid diet-induced liver damage were studied. The findings can provide good evidence for the utilization of TIK as natural antioxidants for the prevention and management of ALD.

## 2. Materials and Methods

### 2.1. Materials and Reagents

**Figure 1:**
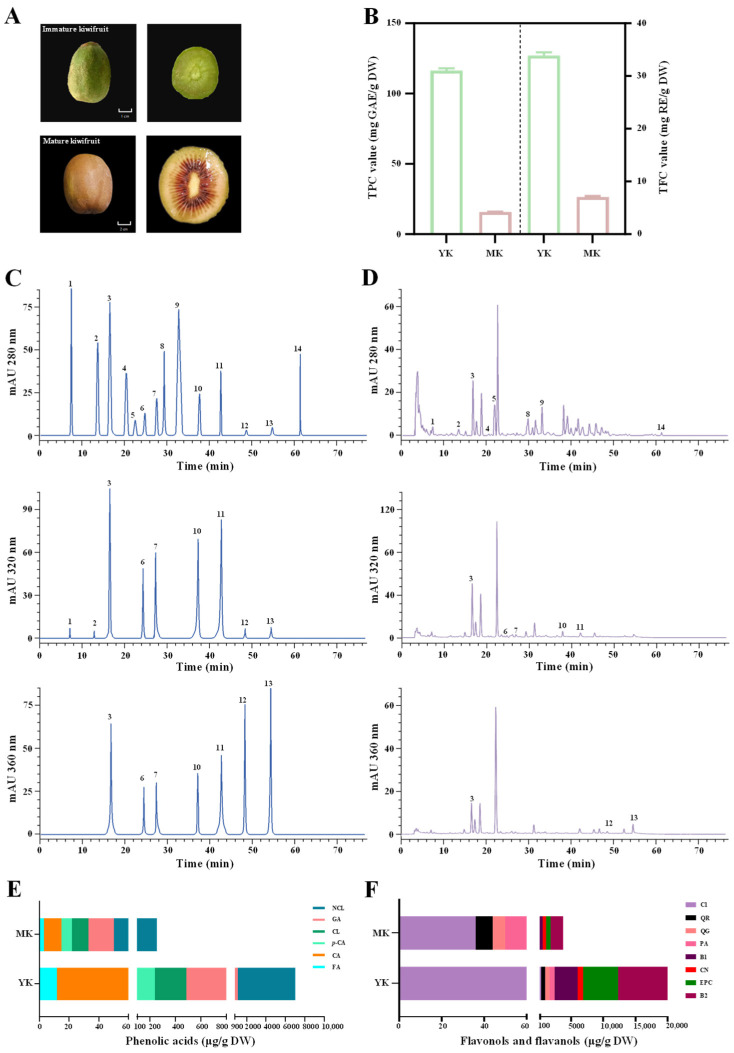
Morphological characteristics of thinned immature and mature kiwifruits (**A**), total polyphenols in their polyphenol-enriched extracts (**B**), HPLC chromatograms of mixed phenolic standards (**C**) and polyphenol-enriched extract from thinned immature kiwifruits (**D**), and levels of individual phenolic compounds in thinned immature and mature kiwifruits (**E**,**F**). YK and MK indicate polyphenol-enriched extracts from thinned immature kiwifruits and mature kiwifruits, respectively; Compounds 1–14 were gallic acid (GA), protocatechuic acid (PA), neochlorogenic acid (NCL), procyanidin B1 (B1), catechin (CN), chlorogenic acid (CL), caffeic acid (CA), procyanidin B2 (B2), epicatechin (EPC), p-coumaric acid (p-CA), ferulic acid (FA), quercetin 3-O-glucoside (QG), quercetin 3-O-rhamnoside (QR), and procyanidin C1 (C1), respectively.

As shown in Figure 1A, TIK and the corresponding mature fruits of *A. chinensis* cv. ‘Hongao’ were collected from a Sichuan Deyang kiwifruit breeding and planting base (GPS coordinates 104°9′17″° E, 31°23′47″° N). Specifically, TIK were collected at 20 days after fruit-setting, while mature fruits were collected at 110 days after fruit-setting.

Polyphenol standards, including chlorogenic acid (CL, B20782), neochlorogenic acid (NCL, B21396), caffeic acid (CA, B20660), ferulic acid (FA, B20007), gallic acid (GA, B20851), p-coumaric acid (p-CA, B20335), protocatechuic acid (PA, B21614), catechin (CN, B21722), epicatechin (EPC, B20102), procyanidin C1 (C1, B50687), procyanidin B1 (B1, B21616), procyanidin B2 (B2, B21617), quercetin 3-O-rhamnoside (QR, B20526), and quercetin 3-O-glucoside (QG, B21529), were purchased from Shanghai Yuanye Biotechnology CO., Ltd. (Shanghai, China). ELISA kits for the determination of proinflammatory cytokines were obtained from Wuhan Elabscience Biotechnology Co., Ltd. (Wuhan, China). Assay kits for the detection of alkaline phosphatase (ALP), aspartate aminotransferase (AST), alanine aminotransferase (ALT), triglyceride (TG), total cholesterol (TC), superoxide dismutase (SOD), catalase (CAT), glutathione (GSH), and malondialdehyde (MDA) were obtained from Nanjing Jiancheng Bioengineering Institute Co., Ltd. (Nanjing, China). The Lieber–DeCarli ethanol liquid diet was obtained from Xiaoshu Youtai (Beijing) Biotechnology Co., Ltd. (Beijing, China).

### 2.2. Preparation of Polyphenol-Enriched Extracts from Thinned Immature and Mature Kiwifruits

Polyphenolic extracts from TIK and mature fruits were extracted by ultrasound-assisted deep eutectic solvent extraction (UDEE) according to a previously developed method in our research group [11]. After the UDEE, the D101 macroporous resin was applied for the fractionation of polyphenol-enriched extracts following our previously established methods [12]. Afterward, polyphenol-enriched extracts from TIK and mature fruits were obtained, and coded as YK and MK, respectively. Finally, one proportion of polyphenol-enriched extract was directly utilized for the determination of total polyphenols, major phenolic compounds, and in vitro antioxidant and anti-inflammatory activities. Another part of polyphenol-enriched extracts was freeze-dried and then utilized for the evaluation of hepatoprotective effects on alcohol-induced liver damage. Detailed methods for the extraction and fractionation of polyphenol-enriched extracts from TIK and mature fruits are supplied in the Supplementary Materials (Section S.1).

### 2.3. Determination of Total Polyphenols and Major Phenolic Compounds in Polyphenol-Enriched Extracts

The levels of total polyphenols in polyphenol-enriched extracts from TIK and mature fruits were measured by colorimetric methods in accordance with our previous study [11]. In brief, the level of total phenolic content (TPC) was measured by the Folin–Ciocalteu colorimetric method, which was expressed in mg GAE/g DW. In addition, the level of total flavonoid content (TFC) was measured by the AlCl_3_-based colorimetric method, which was expressed in mg RE/g DW. Furthermore, the main phenolic components in polyphenol-enriched extracts were determined using an Agilent 1260 II HPLC system according to our previously established methodology [13]. Fourteen commercial standards, including six phenolic acids, six flavanols, and two flavonols, were determined. The level of each phenolic component was expressed in micrograms per gram fruit dry weight (μg/g DW). The detailed methods for the measurement of total polyphenols and major phenolic components in polyphenol-enriched extracts from TIK and mature fruits are supplied in the Supplementary Materials (Sections S.2 and S.3).

### 2.4. Evaluation of In Vitro Antioxidant Activities of Polyphenol-Enriched Extracts

To assess the in vitro antioxidant capacities of polyphenol-enriched extracts from TIK and mature fruits, their scavenging abilities against ABTS, DPPH, and hydroxyl (OH) radicals as well as ferric-reducing antioxidant powers (FRAP) were determined following previously developed methods [11,13]. The levels of FRAP were expressed as micromolar Trolox equivalents per gram fruit dry weight (µmol Trolox/g DW). In addition, the IC_50_ values for free radical scavenging abilities were presented as mg fruit dry weight per mL (mg DW/mL). The detailed methods for evaluating the in vitro antioxidant activities of polyphenol-enriched extracts from TIK and mature fruits are supplied in the Supplementary Materials (Section S.4.1).

### 2.5. Evaluation of In Vitro Anti-Inflammatory Activities

A lipopolysaccharide (LPS)-induced RAW 264.7 cell model was used to determine the in vitro anti-inflammatory effects of polyphenol-enriched extracts from TIK and mature fruits following previous studies with minor modifications [11,14]. The inhibitory effects of polyphenol-enriched extracts from TIK and mature fruits on the release of proinflammatory factors from LPS-induced RAW 264.7 cells, e.g., NO, IL-6, and TNF-α, were evaluated following the manufacturers’ protocols. Detailed methods for the determination of anti-inflammatory effects of polyphenol-enriched extracts from TIK and mature fruits are supplied in the Supplementary Materials (Section S.4.2).

### 2.6. Ameliorative Effects of Polyphenol-Enriched Extracts on Alcoholic Liver Disease in Mice

#### 2.6.1. Animals and Experimental Design

All procedures regarding animal studies were conducted in compliance with the guidelines and regulations of ’the instructive notions with respect to caring for laboratory animals’ issued by the Ministry of Science and Technology of the People’s Republic of China, and received approval from the Ethics Committee on the Use and Care of Animals of Chengdu University (Approval No. EAEC-20118). Forty-two C57BL/6J mice (male, 8-week-old, 22 ± 2 g) were obtained from Beijing Spelford Biotech. Co., Ltd. (Beijing, China). All mice were housed at 25 ± 1 °C under a 12 h light–dark cycle, with free access to water and feed.

The National Institute on Alcohol Abuse and Alcoholism (NIAAA) mice model of alcoholic liver disease (ALD) was used in this study, which was conducted following previous studies with minor modifications [15,16,17]. In brief, after seven days of acclimation, forty-two C57BL/6J mice were randomly divided into 7 groups as follows: control group (CK), model group (MD), positive control group (100 mg/kg of silymarin, PC), mature fruit group with a high dose (2.0 g/kg of polyphenol-enriched extract, MKH), mature fruit group with a low dose (1.0 g/kg of polyphenol-enriched extract, MKL), thinned immature fruit group with a high dose (2.0 g/kg of polyphenol-enriched extract, YKH), and thinned immature fruit group with a low dose (1.0 g/kg of polyphenol-enriched extract, YKL). The MD and CK groups were orally administered with deionized water every day during the experimental period. The PC group was orally administered with 100 mg/kg of silymarin every day during the experimental period. The MKH, MKL, YKH, and YKL groups were orally administered with corresponding polyphenol-enriched extracts every day during the experimental period. The CK group was fed the control Lieber–DeCarli liquid diet, and all other groups were fed a Lieber–DeCarli ethanol liquid diet with 3% ethanol from the 8th day to the 10th day, with 5% ethanol from the 11th day to the 12th day, and with 6% ethanol from the 13th day to the 22nd day, respectively. On the 23rd day, mice in the control group were orally administered with maltose dextrin, and mice in all other groups were orally administered with 30% of ethanol in the early morning. After 9 h, all mice were euthanized, and liver tissues and blood samples were collected.

#### 2.6.2. Histological Analysis and Biochemical Assays

The left lobe livers of all mice were fixed with 4% paraformaldehyde, embedded with paraffin, and then cut into 4 μm thick sections for hematoxylin and eosin (H&E) staining. Additionally, the rest of the liver tissues were homogenized by adding saline at a ratio of 1:9 (g/mL). Afterward, the mixture was centrifuged at 2500 rpm for 10 min, and the resulting supernatant was collected. The levels of TG, TC, GSH, MDA, SOD, and CAT in the supernatant were measured using the commercial kits following the manufacturer’s instructions. The levels of hepatic inflammatory factors, including IL-β, IL-6, and TNF-α, were measured using ELISA kits following the manufacturer’s instructions. Furthermore, blood samples were centrifuged immediately at 3000 rpm for 10 min to obtain the supernatant plasma. The levels of serum AST, ALT, and ALP were assessed using the commercial kits following the manufacturer’s instructions.

### 2.7. Statistical Analysis

SPSS 22.0 software (SPSS Inc., Chicago, IL, USA) was applied for data analysis. Statistical analysis was conducted using one-way ANOVA or a two-tailed Student’s *t*-test, and *p* < 0.05 was considered to be statistically significant.

## 3. Results and Discussion

### 3.1. Comparison of Total Polyphenols and Individual Phenolic Components in Thinned Immature and Mature Kiwifruits

Polyphenols are regarded as the most vital components in kiwifruits, which are responsible for their various health-promoting benefits [8,18,19]. In fact, our recent findings have shown that thinned unripe fruits from red-fleshed kiwifruits possess plentiful polyphenols, such as PA, CN, EPC, B1, and B2 [11]. As displayed in Figure 1B, the present findings further confirmed that thinned immature fruits from red-fleshed kiwifruits were much richer in total polyphenols than their corresponding mature fruits. In particular, the TPC of thinned immature fruits (116.39 ± 1.51 mg GAE/g DW) was approximately 7.4 times greater than that of their corresponding mature fruits (15.75 ± 0.29 mg GAE/g DW). Similarly, the TFC of thinned immature fruits (33.88 ± 0.59 mg RE/g DW) was about 4.8 times higher than that of mature fruits (7.05 ± 0.24 mg RE/g DW). The present findings are similar to an earlier study that observed that total polyphenols in thinned green and yellow fleshed immature kiwifruits were higher than those of their corresponding mature fruits [10]. Furthermore, based on our earlier studies [11,13], fourteen commercially available phenolic compound standards were measured in thinned immature and mature fruits. In particular, six phenolic acids (NCL, GA, CL, p-CA, CA, and FA), six flavanols (C1, PA, B1, CN, EPC, and B2), and two flavonols (QG and QR) were detected in thinned immature and mature fruits from red-fleshed kiwifruit. Figure 1C, D displays the HPLC profiles of phenolic standards and polyphenol-enriched extract from TIK, detected at 280 nm, 320 nm, and 360 nm, respectively. In addition, their levels in thinned immature fruits and mature fruits are displayed in Figure 1E,F, and their detailed contents are summarized in Table 1. As displayed in Figure 1E, the level of total phenolic acids in polyphenol-enriched extract from TIK was obviously higher than that of mature fruits, similar to the levels of TPC observed in immature fruits and mature fruits (Figure 1B). In addition, as displayed in Figure 1F, the levels of total flavonols and total flavanols in polyphenol-enriched extract from TIK were also obviously greater than those of mature kiwifruits, similar to the levels of TFC observed in immature and mature kiwifruits (Figure 1B). In detail, NCL was detected as the predominant phenolic acid in both thinned immature and mature kiwifruits, with levels of 6209.89 μg/g DW and 314.46 μg/g DW, respectively. The present finding was comparable to earlier studies which that observed both immature and mature fruits from red-fleshed kiwifruits were rich in NCL [11,13,20]. In addition, B2, B1, and EPC were detected as the major flavanols, even the predominant phenolic components, in both thinned immature and mature kiwifruits, which was comparable to earlier studies [10,11,13]. In particular, B2 was observed as the most abundant flavanol in both thinned immature and mature kiwifruits, with levels of 9857.14 μg/g DW and 1971.01 μg/g DW, respectively. Furthermore, two flavonols, including QG and QR, were detected in both thinned immature and mature kiwifruits, with levels ranging from 67.21 μg/g DW to 820.29 μg/g DW, and from 85.90 μg/g DW to 521.63 μg/g DW, respectively. Collectively, these findings clearly demonstrate that TIK are promising resources of valuable polyphenolics, which hold great potential to be developed into value-added functional products.

### 3.2. Comparison of In Vitro Antioxidant and Anti-Inflammatory Activities of Thinned Immature and Mature Kiwifruits

**Figure 2:**
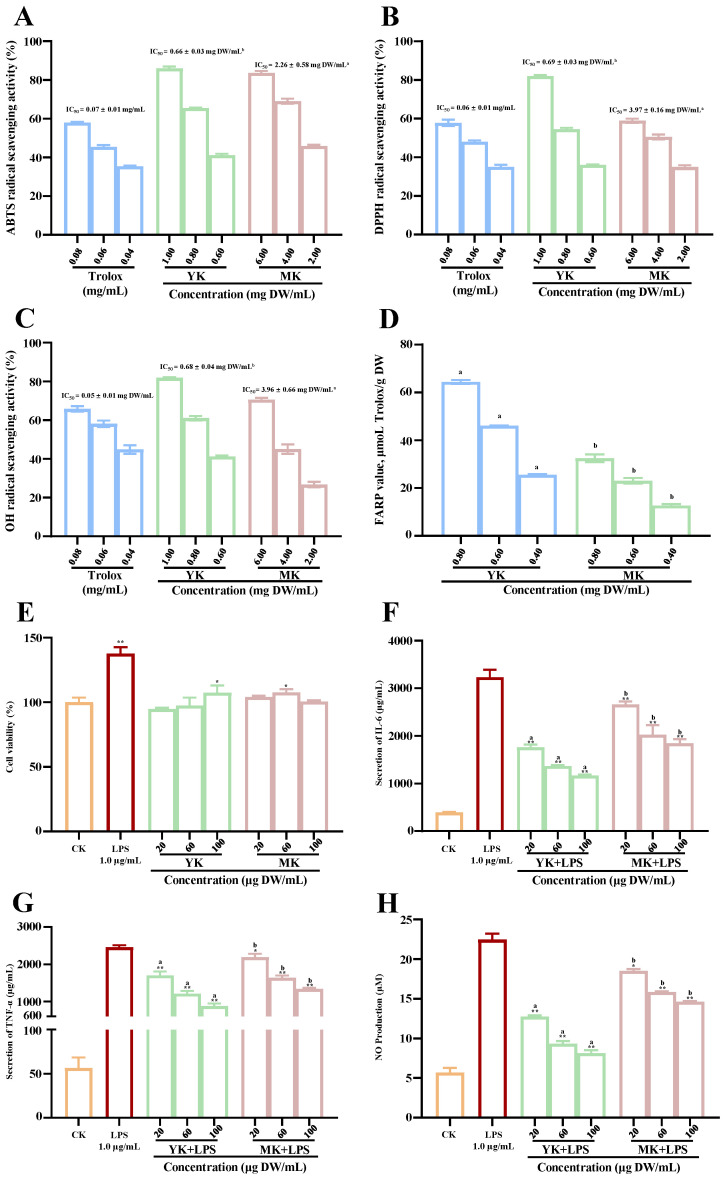
In vitro antioxidant capacities (**A**–**D**) and anti-inflammatory effects (**E**–**H**) of polyphenol-enriched extracts from thinned immature and mature kiwifruits. A, B, and C correspond to ABTS, DPPH, and OH radical scavenging abilities; D, ferric-reducing antioxidant power; E, cell viability of RAW 264.7 cells; F, secretion of IL-6 from LPS-stimulated RAW 264.7 cells; G, secretion of TNF-α from LPS-stimulated RAW 264.7 cells; H, NO production from LPS-stimulated RAW 264.7 cells; YK and MK indicate polyphenol-enriched extracts from thinned immature kiwifruits and mature kiwifruits, respectively; different letters (a–b) indicate statistically significant differences (*p* < 0.05) between thinned immature kiwifruits and mature kiwifruits; significant differences in cell viability of LPS and kiwifruit extracts vs. control are shown by * *p* < 0.05 and ** *p* < 0.01. Significant differences in NO production, secretion of IL-6, and secretion of TNF-α in kiwifruit extracts vs. LPS are shown by ** *p* < 0.01.

Generally, ALD is closely correlated to oxidative stress and inflammatory reaction [1,2,3,21,22], and the reduction in hepatic oxidative stress and inflammatory reaction can effectively regulate the development of ALD. In fact, it is well established that natural polyphenols are strong antioxidants which can alleviate hepatic oxidative stress, hepatic inflammation, and fat accumulation induced by acute or chronic alcohol consumption [4,7,23]. Therefore, to assess the potential protective effect of polyphenol-enriched extracts from thinned immature and mature kiwifruits against ALD, their in vitro antioxidant capacities and anti-inflammatory activities were systematically investigated and compared. As displayed in Figure 2A–D, polyphenol-enriched extracts from both thinned immature and mature fruits from red-fleshed kiwifruit exerted remarkable in vitro antioxidant capacities; they could effectively scavenge various free radicals and exhibited strong FRAP values. In particular, the in vitro antioxidant capacities of polyphenol-enriched extract from thinned immature fruits were much stronger than those of mature fruits, which could be attributed to their much higher levels of total polyphenols, comparable to earlier studies where the antioxidant capacity of kiwifruit was mainly attributed to its high content of polyphenols [10,24,25,26]. In detail, the IC_50_ levels of polyphenol-enriched extract from thinned immature fruits against the ABTS, DPPH, and OH radicals were measured to be 0.66, 0.69 L, and 0.68 mg DW/mL, respectively, which were much lower than for those of polyphenol-enriched extract from their corresponding mature fruits, with IC_50_ levels of 2.26, 3.97, and 3.96 mg DW/mL, respectively. Furthermore, the FRAP values of polyphenol-enriched extracts from thinned immature fruits and mature fruits were measured to be 64.44 µmol Trolox/g DW and 32.47 µmol Trolox/g DW with a concentration of 0.8 mg DW/mL, respectively. Overall, these results implied that these discarded immature kiwifruits are potential resources of natural antioxidants, and might effectively regulate the development of ALD by reducing oxidative stress in the liver.

Furthermore, as displayed in Figure 2E, polyphenol-enriched extracts from both TIK and mature kiwifruits exhibited no cytotoxic effects on RAW 264.7 cells. However, polyphenol-enriched extracts from both TIK and mature kiwifruits could significantly affect the production of inflammatory factors (NO, IL-6, and TNF-α) from LPS-stimulated RAW 264.7 cells (Figure 2F–H), thereby exhibiting potential in vitro anti-inflammatory activities. Notably, polyphenol-enriched extract from thinned immature fruits exhibited significantly stronger anti-inflammatory activity than that of mature kiwifruits, which was also attributed to their higher level of total polyphenols and phenolic compounds. This can be attribute to the well-established fact that polyphenols, e.g., procyanidins and phenolic acids, possess remarkable anti-inflammatory effects [27,28,29,30]. Overall, these findings implied that polyphenol-enriched extract from TIK could be utilized for the amelioration of chronic inflammatory diseases, and could be potential functional ingredients for the improvement of ALD via reducing hepatic inflammatory reaction in the liver.

### 3.3. Comparison of Protective Effects of Polyphenol-Enriched Extracts from Thinned Immature and Mature Kiwifruits against Alcoholic Liver Disease in Mice

#### 3.3.1. Effects of Polyphenol-Enriched Extracts on Alcohol-Induced Liver Injury in Mice

**Figure 3:**
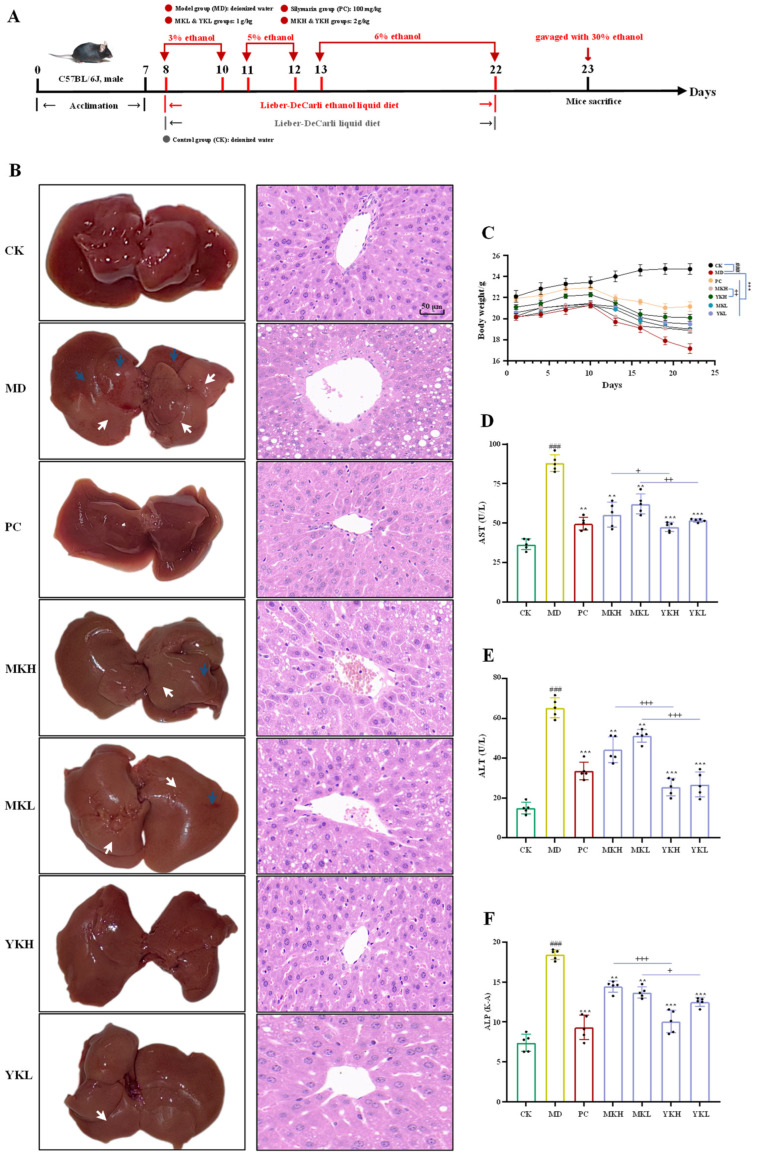
The protective effects of polyphenol-enriched extracts from thinned immature and mature kiwifruits against alcoholic liver disease model mice. (**A**) An overview of the model procedures; (**B**) morphological characteristics and H&E staining of mice liver in each group (×400 magnification); (**C**) body weight growth of mice during the experimental period; (**D**–**F**) levels of AST, ALT, and ALP in liver tissue (n = 5); significant differences in the CK group vs. MD group are shown by ### *p* < 0.001; significant differences in sample groups vs. the MD group are shown by ** *p* < 0.01 and *** *p* < 0.001; significant differences between the thinned immature kiwifruit group and mature kiwifruit group are shown by + *p* < 0.05, ++ *p* < 0.01, and +++ *p* < 0.001.

To assess the hepatoprotective effects of polyphenol-enriched extracts from thinned immature and mature kiwifruits on alcohol-induced liver damage, the Lieber–DeCarli ethanol liquid diet-induced alcoholic liver disease model was utilized in this study (Figure 3A). Figure 3B shows the morphological characteristics and H&E staining of liver tissues. Obviously, the liver tissue of the CK group exhibited a bright red appearance and smooth surface, while the liver tissue of the MD group exhibited notable hyperaemia and whitish patches. Nevertheless, after the treatment of polyphenol-enriched extract from thinned immature kiwifruits (YK) or mature kiwifruits (MK), the pathological appearance of liver tissue was obviously improved, especially with the administration of a high-dose of YK (YKH group, 2.0 g/kg of YK). In addition, the H&E staining analysis showed that the liver tissue of the MD group exhibited various histopathological changes, e.g., unevenly arranged hepatocytes, a high level of fat droplets and fatty vacuoles, lots of necrotic cells, and inflammatory infiltration. Nevertheless, the administration of YK or MK could obviously ameliorate the hepatic histopathology; in particular, the liver tissue in the YKH group was comparable to that of the CK group, while a small number of fat droplets and fatty vacuoles could still be observed in the liver tissue of the MKH group (2.0 g/kg of MK). These findings implied that the hepatoprotective effect of YK on alcohol-induced liver damage was stronger than that of MK. Furthermore, as displayed in Figure 3C, the body weight of mice in the MD group significantly decreased in comparison with the CK group, while the administration of YK or MK significantly reversed the body weight loss. In particular, the recovery of body weight in the YKH group was much better than that of the MKH group. These findings implied that the hepatoprotective effect of YK on alcohol-induced liver damage was better than that of MK at both low and high doses.

**Figure 4:**
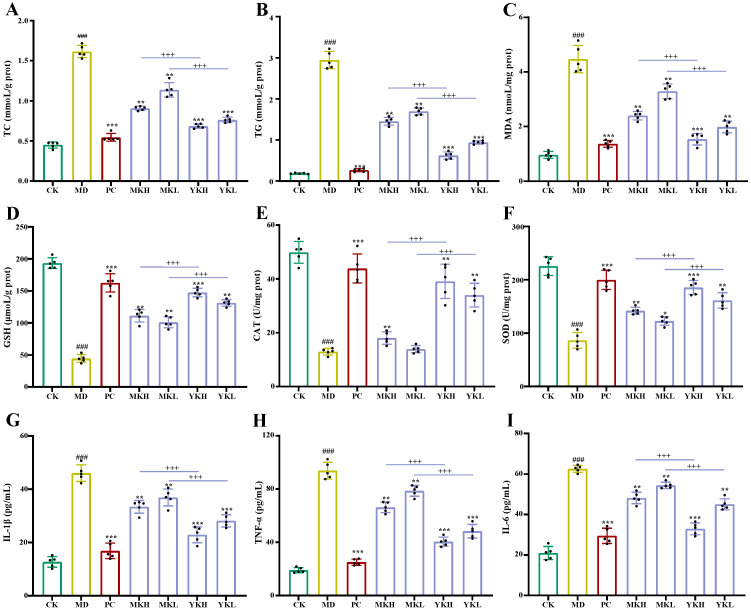
The levels of biochemical indicators TC (**A**), TG (**B**), MDA (**C**), GSH (**D**), CAT (**E**), and SOD (**F**) and proinflammatory factors IL-1β (**G**), I TNF-α (**H**), and IL-6 (**I**) in mice liver tissues (n = 5). Significant differences in the CK group vs. MD group are shown by ### *p* < 0.001; significant differences in sample groups vs. the MD group are shown by * *p* < 0.05, ** *p* < 0.01, and *** *p* < 0.001; significant differences between the thinned immature kiwifruit group and mature kiwifruit group are shown by +++ *p* < 0.001.

Generally, AST, ALT, and ALP are crucial markers for the assessment of liver function, which are sensitive indicators of liver injury and the loss of membrane functional integrity [31,32]. When the liver function is damaged, the serum levels of AST, ALT, and ALP will be significantly elevated. As displayed in Figure 3D–F, the serum levels of AST, ALT, and ALP in the MD group significantly increased when compared with the CK group, implying that the liver was damaged by ethanol. Conversely, the administration of MK or YK could significantly lower the serum levels of AST, ALT, and ALP, suggesting that both MK and YK possessed potential protective effects for ALD. Additionally, the serum levels of AST, ALT, and ALP in the YKH group and YKL group were significantly lower than those of the MKH group and MKL group, respectively, further implying that the hepatoprotective effect of polyphenol-enriched extract from TIK was better than that of mature fruit. Moreover, to assess the beneficial effects of both YK and MK on ethanol-induced hepatic steatosis in mice, the levels of TG and TC in the liver were determined. Generally, the long-term consumption of ethanol can lead to the increase in serum and hepatic TG and TC concentrations in mice [7]. As displayed in Figure 4A,B, the levels of hepatic TC and TG in the MD group significantly improved when compared with the CK group. However, after the treatment of MK or YK, the levels of hepatic TG and TC significantly declined, indicating that both MK and YK could ameliorate hepatic fat accumulation. In addition, the levels of hepatic TG and TC in the YKH group and YKL group were significantly lower than those of the MKH group and MKL group, respectively, further confirming that polyphenol-enriched extract from thinned immature fruits exhibited better beneficial effects on alcohol-induced hepatic steatosis than that of mature fruits. Overall, the administration of polyphenol-enriched extracts from both immature and mature kiwifruits exerted potential hepatoprotective effects on alcohol-induced liver damage, and the polyphenol-enriched extract from thinned immature fruits was more effective than that of mature kiwifruits.

#### 3.3.2. Effects of Polyphenol-Enriched Extracts on Ethanol-Induced Oxidative Stress in Mice

During alcohol metabolism, various reactive oxygen species (ROS) are generated in the liver, which can cause oxidant system imbalance, thereby leading to oxidative stress, hepatocellular damage, and lipid peroxidation [2,4,33]. Oxidative stress plays a critical role in the development of ALD [4]. Therefore, enhancing the antioxidant protective capacity of the liver is regarded as a promising approach for the management of ALD. Owing to the excellent in vitro antioxidant capacities of polyphenol-enriched extracts from TIK and mature fruits, their effects on ethanol-induced oxidative stress in mice were further investigated to better understand their protective effects on ALD.

Generally, the long-term consumption of alcohol can increase the level of hepatic MDA, which can promote lipid peroxidation and enhance oxidative stress [34]. As displayed in Figure 4C, the level of hepatic MDA in the MD group was significantly increased. Conversely, after the administration of MK or YK, the levels of hepatic MDA significantly decreased, indicating that both MK and YK could ameliorate oxidative stress by inhibiting the generation of MDA in the liver. Notably, the inhibitory effects of YK were stronger than those of MK, which were probably attributed to the stronger in vitro antioxidant capacities of YK. Furthermore, as opposed to hepatic MDA, both enzymatic and nonenzymatic antioxidant defense systems (e.g., SOD, CAT, GSH-Px, and GSH) in the liver can clear ROS to reduce oxidative stress [7]. As displayed in Figure 4D–F, the levels of GSH, CAT, and SOD in liver tissues of the MD group significantly decreased when compared with the CK group. Nevertheless, the administration of both MK and YK could significantly promote the levels of GSH, CAT, and SOD in liver tissues, respectively, implying that both MK and YK could ameliorate oxidative stress by enhancing antioxidant enzymatic activities. Specifically, once again, the beneficial effect of YK on ethanol-induced oxidative stress in mice was much better than that of MK, which was probably attributed to its higher level of antioxidant polyphenols and individual phenolic compounds, e.g., B1, B2, and NCL. Overall, these results suggested that polyphenol-enriched extract from TIK could ameliorate ALD in mice via restoring the levels of antioxidant enzymes in the liver.

#### 3.3.3. Effects of Polyphenol-Enriched Extracts on Ethanol-Induced Inflammation in Mice

In addition to hepatic oxidative stress, the inflammatory response also plays a crucial role in the pathogenesis of ALD [7,35,36]. Generally, alcohol consumption can activate innate and adaptive immunity to generate a lot of cytokines and chemokines in the liver, thereby causing neutrophil infiltration to trigger alcoholic hepatitis [37]. Furthermore, long-term alcohol consumption may cause intestinal barrier damage, leading to LPS reaching the liver [6,35,38]. This gut-derived LPS can activate the TLR 4-NF-κB pathway to release proinflammatory cytokines, ultimately causing liver inflammation [3]. Hence, the intervention of hepatic inflammation can also improve the development of ALD. As displayed in Figure 4G–I, the proinflammatory cytokines in the MD group were significantly up-regulated in comparison to those of the CK group. Nevertheless, the administration of MK or YK could notably down-regulate the levels of IL-1β, TNF-α, and IL-6 in the liver tissues, suggesting that both MK and YK could ameliorate ALD via regulating hepatic inflammation. In particular, the inhibitory effects of YK on hepatic inflammation were significantly higher than those of MK, which were comparable to their in vitro anti-inflammatory effects. The stronger beneficial effect of YK on ethanol-induced inflammation in mice is probably attributable to its higher content of polyphenols. In fact, it is well established that polyphenols can improve the development of ALD via regulating hepatic inflammation [4,7]. Overall, these results suggested that polyphenol-enriched extract from TIK could also ameliorate ALD in mice via down-regulating hepatic inflammatory responses.

## 4. Conclusions

In this study, phenolic compounds from TIK and their corresponding mature fruits were investigated, and their protective effects against ethanol-induced ALD in mice were studied. The findings revealed that TIK had a much higher level of valuable phenolic compounds than that of their corresponding mature fruits, with NCL, EPC, B1, and B2 detected as the major polyphenols. As a consequence, TIK exhibited much stronger in vitro antioxidant capacities and anti-inflammatory activities than those of mature fruits. Most importantly, our findings demonstrated that both polyphenol-enriched extracts from TIK and their corresponding mature fruits could notably ameliorate ALD in mice by exerting antioxidant and anti-inflammatory effects on the liver. In particular, compared with mature fruits, polyphenol-enriched extracts from TIK exerted superior protective effects against ALD, which is probably attributable to its much higher levels of polyphenols. Collectively, the findings suggest that TIK are potential resources of natural antioxidants, which can be developed as value-added functional products for the intervention of ALD.

## Figures and Tables

**Table 1:** Calibration data of fourteen phenolic components and their levels in thinned immature and mature kiwifruits.

Compounds	Regression Equation	R^2^	Linear Range (μg/mL)	YK	MK
GA	y = 14.9049x − 45.5985	0.9942	0.56–71.43	652.92 ± 23.31 ^a^	19.25 ± 0.99 ^b^
PA	y = 12.812 − 30.4955	0.9957	0.56–71.43	713.05 ± 22.70 ^a^	51.22 ± 1.54 ^b^
NCL	y = 30.4434x − 172.967	0.9983	5.73–71.43	6209.89 ± 197.53 ^a^	314.46 ± 7.71 ^b^
B1	y = 4.6754x − 9.235	0.9991	5.73–71.43	3115.16 ± 141.69 ^a^	731.49 ± 25.88 ^b^
CN	y = 10.972x − 61.241	0.9987	5.73–71.43	921.14 ± 42.91 ^a^	509.74 ± 22.67 ^b^
CL	y = 19.5361x − 109.581	0.9964	5.73–71.43	227.76 ± 10.41 ^a^	7.92 ± 0.22 ^b^
CA	y = 137.59x − 63.175	0.9947	5.73–71.43	78.69 ± 3.31 ^a^	23.79 ± 1.04 ^b^
B2	y = 9.89x − 17.1126	0.9963	5.73–71.43	9857.14 ± 384.42 ^a^	1971.01 ± 87.66 ^b^
EPC	y = 9.184x − 30.653	0.9974	5.73–71.43	5890.79 ± 153.76 ^a^	1638.87 ± 29.57 ^b^
*p*-CA	y = 217.0018x − 119.512	0.9970	0.56–71.43	181.78 ± 2.99 ^a^	8.89 ± 0.34 ^b^
FA	y = 203.622x − 22.17	0.9984	0.56–71.43	15.59 ± 0.71 ^a^	5.12 ± 0.22 ^b^
QG	y = 51.0929x − 33.0198	0.9959	0.56–71.43	820.29 ± 13.71 ^a^	67.21 ± 1.15 ^b^
QR	y = 33.099x − 93.358	0.9963	2.85–15.18	521.63 ± 1.66 ^a^	85.90 ± 2.31 ^b^
C1	y = 206.91x − 517.43	0.9960	1.20–14.28	352.36 ± 5.15 ^a^	61.72 ± 1.25 ^b^
Total content (μg/g DW)				29,558.19 ± 1170.58 ^a^	5496.59 ± 174.86 ^b^

YK and MK indicate polyphenol-enriched extracts from thinned immature kiwifruits and mature kiwifruits, respectively; GA, gallic acid; PA, protocatechuic acid; NCL, neochlorogenic acid; B1, procyanidin B1; CN, catechin; CL, chlorogenic acid; CA, caffeic acid; B2, procyanidin B2; EPC, epicatechin; p-CA, p-coumaric acid; FA, ferulic acid; QG, quercetin 3-O-glucoside; QR, quercetin 3-O-rhamnoside; C1, procyanidin C1; different letters in the same column indicate significant differences at *p* < 0.05.

## Data Availability

The original contributions presented in the study are included in the article/Supplementary Materials, further inquiries can be directed to the corresponding authors.

## Supplementary Materials

The following supporting information can be downloaded at https://www.mdpi.com/article/10.3390/foods13193072/s1, Section S.1: Preparation of polyphenol-enriched extracts from thinned immature kiwifruits and mature kiwifruits; Section S.2: Determination of total polyphenols in polyphenol-enriched extracts from thinned immature kiwifruits and mature kiwifruits; Section S.3: HPLC analysis of major phenolic compounds in polyphenol-enriched extracts from thinned immature kiwifruits and mature kiwifruits; Section S.4: Evaluation of antioxidant and anti-inflammatory activities of polyphenol-enriched extracts from thinned immature kiwifruits and mature kiwifruits.
