# Supplementary material for: Comparison of Protective Effects of Polyphenol-Enriched Extracts from Thinned Immature Kiwifruits and Mature Kiwifruits against Alcoholic Liver Disease in Mice
Source: Foods. 2024 Sep 26;13(19):3072. doi: 10.3390/foods13193072 (PMC11475074; doi:10.3390/foods13193072)
Supplement: Supplementary file 1 [file foods-13-03072-s001.zip › foods-3204068-supplementary.pdf]

# Comparison of protective effects of polyphenol-enriched extracts from thinned immature kiwifruits and mature kiwifruits against alcoholic liver disease in mice

Wen Deng [1,2], Qian-Ni Yang [1,2], Ding-Tao Wu [2,*], Jie Li [3], Hong-Yan Liu [3],

Yi-Chen Hu [1], Liang Zou [1], Ren-You Gan [4], Hui-Ling Yan [1], Jing-Wei Huang [1,*]

[1] Key Laboratory of Coarse Cereal Processing of Ministry of Agriculture and Rural Affairs, School of Food and Biological Engineering, Chengdu University, Chengdu 610106, Sichuan, China

[2] Institute for Advanced Study, Chengdu University, Chengdu 610106, Sichuan, China

[3] Research Center for Plants and Human Health, Institute of Urban Agriculture, Chinese Academy of Agricultural Sciences, National Agricultural Science and Technology Center, Chengdu 610213, Sichuan, China

[4] Department of Food Science and Nutrition, Faculty of Science, The Hong Kong Polytechnic University, Kowloon, Hong Kong, China

[*] Corresponding authors

E-mail addresses: wudingtao@cdu.edu.cn (D.-T.W.); huangjingwei@cdu.edu.cn (J.-W.H.)

**Section S.1 Preparation of polyphenol-enriched extracts from thinned immature kiwifruits and mature kiwifruits**

Polyphenol-enriched extracts from thinned immature kiwifruits and mature kiwifruits were prepared by ultrasound-assisted deep eutectic solvent extraction (UDEE). In detail, the deep eutectic solvent (DES) was formulated by mixing choline chloride and glycerol in a molar ratio of 1: 2, followed by the addition of 32% (*v/v*) of water under gentle stirring. The mixture was then sonicated by an ultrasonic cleaning cell until a homogenous solution was obtained. 5.0 g of each kiwifruit powder was mixed with 250 mL of DES extraction solvent, and extracted using a JY92-IIN ultrasonic processor (Ningbo Scientz Biotechnology Co., Ltd., Ningbo, China). The ultrasonic extraction power and ultrasonic extraction time were set as 450 W and 23 min, respectively. The polyphenol-enriched extract was then subjected to centrifugation (6000 × g, 4 °C, 15 min), and the supernatant was further isolated by D101 macroporous resin. A column (60.6 mm × 218.5 mm) was slurry-packed with 400 g of D101 macroporous resin in the deionized water, and then the column was sequentially washed with ethanol, 3% of HCl, 3% of NaOH, and deionized water. The concentrated supernatant was loaded onto the column, and then eluted with water and 60% of methanol (*v/v*), with the flow rate of 30 mL/min. The eluted fractions from 60% of methanol were collected, and concentrated to obtain polyphenol-enriched extracts from thinned immature kiwifruits and mature kiwifruits, which were coded as YK and MK, respectively. Furthermore, one proportion of polyphenol-enriched extracts was directly utilized for the determination of polyphenols and major phenolic compounds and for

the evaluation of *in vitro* antioxidant and anti-inflammatory activities. Another part of polyphenol-enriched extracts was freeze-dried for the evaluation of protective effects against alcoholic liver injury in mice.

**Section S.2 Determination of total polyphenols in polyphenol-enriched extracts from thinned immature kiwifruits and mature kiwifruits**

The contents of total polyphenols, including total phenolic content (TPC) and total flavonoid content (TFC), were determined by colorimetric methods. In detail, the levels of TPC in thinned immature kiwifruits and mature kiwifruits were determined using a modified Folin-Ciocalteu colorimetric method. A suitably diluted kiwifruit extract (100 μL) was mixed with 500 μL of Folin-Ciocalteu working solution (0.2 M) and reacted at room temperature for 10 min. Afterward, 500 μL of sodium carbonate solution (20%, *w/v*) was added, and the mixture was incubated in the dark for 60 min at room temperature. The absorbance of the mixture was measured at 760 nm with gallic acid as a standard. TPC was expressed as milligram gallic acid equivalent per gram kiwifruit dry weight (mg GAE/g DW).

The levels of TFC in thinned immature kiwifruits and mature kiwifruits were quantified using the $AlCl_3$-based colorimetric method. Briefly, the diluted kiwifruit extract (100 μL) was mixed with 30 μL of $NaNO_2$ (5%, *w/v*) solution and left for 6 min. Then, 30 μL of $Al (NO_3)_3$ (10%, *w/v*) solution was added, and the mixture was reacted for an additional 6 min. Afterward, 400 μL of NaOH (4%, *w/v*) was added, and then the mixture was kept in the dark for 25 min. Finally, the absorbance of the mixture was measured at 510 nm. Rutin was served as a standard for the quantification. TFC was

expressed as milligram rutin equivalent per gram kiwifruit dry weight (mg RE/g DW).

**Section S.3 HPLC analysis of major phenolic compounds in polyphenol-enriched extracts from thinned immature kiwifruits and mature kiwifruits**

The major phenolic compounds in polyphenol-enriched extracts from thinned immature kiwifruits and mature kiwifruits were measured using an Agilent 1260 II HPLC system (Agilent Technologies, Santa Clara, CA, USA). The chromatographic separation was performed at 25 °C using a ZORBAX Eclipse XDB-C18 column (250 mm × 4.6 mm, 5 μm, Agilent Technologies, Santa Clara, CA, USA). The mobile phase was consisted of solvent A (0.5% of acetic acid aqueous solution) and solvent B (acetonitrile). Samples were eluted as follows: 0 min, 5% B; 5 min, 5% B; 50 min, 5-20% B; 70 min, 20-70% B; 72 min, 70-5% B; and 72-77 min, 5% B. The flow rate was 0.8 mL/min, and the injection volume was 20 μL for all samples. Hydroxybenzoic acid and flavanols were determined at 280 nm, hydroxycinnamic acids were measured at 320 nm, and flavonols were detected at 360 nm. In this study, fourteen commercially available phenolic standards, including six phenolic acids (gallic acid, caffeic acid, ferulic acid, *p*-coumaric acid, chlorogenic acid, and neochlorogenic acid), six flavanols (protocatechuic acid, catechin, epicatechin, procyanidin B1, procyanidin B2, and procyanidin C1), and two flavonols (quercetin 3-O-glucoside and quercetin 3-O-rhamnoside), were quantified. The levels of individual phenolic compounds in polyphenol-enriched extracts from thinned immature kiwifruits and mature kiwifruits were expressed as microgram per gram kiwifruit dry weight (μg/g DW).

**Section S.4 Evaluation of antioxidant and anti-inflammatory activities of**

**polyphenol-enriched extracts from thinned immature kiwifruits and mature kiwifruits**

**Section S.4.1 Evaluation of *in vitro* antioxidant activities**

The ABTS radical scavenging ability assay, DPPH radical scavenging ability assay, hydroxyl radical (OH) scavenging ability assay, and ferric-reducing antioxidant power (FRAP) assay were carried out to evaluate *in vitro* antioxidant activities of polyphenol-enriched extracts from thinned immature kiwifruits and mature kiwifruits in this study. For the determination of ABTS radical scavenging ability, the ABTS radical solution was generated by the interaction of 7 mM ABTS solution and 2.45 mM aqueous potassium persulfate at room temperature for at least 16 h in the dark. The ABTS radical solution was then diluted with phosphate buffer (0.2 M, pH 7.4) to an absorbance of 0.780 ± 0.05 at 734 nm. Afterward, 200 μL of ABTS radical working solution was mixed with 20 μL of each kiwifruit extract at three different concentrations (0.06 – 6.00 mg kiwifruit dry weight equivalent per mL, 0.06 – 6.00 mg DW/mL) or phosphate buffer as a negative control in a 96-well microplate to react at 30 °C for 20 min. The absorbance of the mixture was measured at 734 nm, and Trolox was used as a standard. The $IC_{50}$ values were calculated by establishing a logarithmic regression. In addition, for the determination of DPPH radical scavenging ability, 25 μL of each kiwifruit extract at three different concentrations (0.06 – 6.00 mg kiwifruit dry weight equivalent per mL, 0.06 – 6.00 mg DW/mL) or methanol as a negative control was added to 200 μL of DPPH solution (0.35 mM) in a 96-well microplate. The mixed solution was shaken and incubated at room temperature for 30 min. Finally, the absorbance of the

mixture was measured at 517 nm with a blank contain-only DPPH solution and methanol. Trolox was also used as a standard. The $IC_{50}$ values were calculated by establishing a logarithmic regression. Furthermore, for the determination of OH radical scavenging ability, 100 μL of each kiwifruit extract at three different concentrations (0.06 – 6.00 mg kiwifruit dry weight equivalent per mL, 0.06 – 6.00 mg DW/mL) was mixed with 100 μL of $FeSO_4$ (2 mM) and 100 μL of salicylic acid-ethanol solution (6 mM). Afterward, 100 μL of $H_2O_2$ (6 mM) was added to the mixture and then incubated for 30 min at 37 °C in a water bath. The absorbance of the mixture was measured at 510 nm, and the $IC_{50}$ values were calculated by establishing a logarithmic regression. For the determination of FRAP value, the FRAP working solution containing 300 mM acetate buffer (pH 3.6), 10 mM TPTZ solution in 40 mM HCl, and 20 mM FeCl3 solution at a ratio of 10:1:1 was freshly prepared. The FRAP working solution was warmed at 30 °C before usage. Then, 100 μL of each kiwifruit extract at three different concentrations (0.40 – 0.80 mg DW/mL mg kiwifruit dry weight equivalent per mL, 0.40 – 0.80 mg DW/mL) was mixed with 3 mL of FRAP solution and incubated at 37 °C for 4 min. Finally, the absorbance was measured at 593 nm. The levels of FRAP were expressed as micromolar Trolox equivalent per gram kiwifruit dry weight (μmol Trolox/g DW), and $IC_{50}$ values for free radical scavenging abilities were presented as mg kiwifruit dry weight per mL (mg DW/mL).

**Section S.4.2 Evaluation of *in vitro* anti-inflammatory activities**

A lipopolysaccharide (LPS)-induced RAW 264.7 cell model was carried out to evaluate *in vitro* anti-inflammatory activities of polyphenol-enriched extracts from

thinned immature kiwifruits and mature kiwifruits. In brief, the impacts of each kiwifruit extract on the cell viability of RAW 264.7 cells were evaluated by the MTT method, and different concentrations (20 – 100 μg kiwifruit dry weight equivalent per mL, 20 – 100 μg/mL) of each kiwifruit extract were tested. In addition, the RAW 264.7 macrophages were cultivated overnight in 96-well plates at 37 °C with 5% $CO_2$. The culture medium was then replaced with LPS (1 μg/mL) in a volume of 100 μL per well, except for the blank group. After incubating for 24 h, various concentrations (20 – 100 μg kiwifruit dry weight equivalent per mL, 20 – 100 μg/mL) of each kiwifruit extract were added and incubated for an additional 24 h. The supernatant (50.0 μL) was mixed with Griess I (50.0 μL) and Griess II (50.0 μL) reagents at room temperature, and the absorbance was measured at 540 nm. $NaNO_2$ was employed as a reference standard to determine the concentration of NO. The levels of cytokines interleukin-6 (IL-6) and tumor necrosis factor-alpha (TNF-α) in the supernatant were measured by ELISA kits based on the manufacturer’s procedures (Elabscience, Wuhan, China).
